# Advanced Treatment of Pesticide-Containing Wastewater Using Fenton Reagent Enhanced by Microwave Electrodeless Ultraviolet

**DOI:** 10.1155/2015/205903

**Published:** 2015-08-11

**Authors:** Gong Cheng, Jing Lin, Jian Lu, Xi Zhao, Zhengqing Cai, Jie Fu

**Affiliations:** ^1^Shenzhen Academy of Environmental Sciences, Shenzhen 518001, China; ^2^Environmental Engineering Program, Department of Civil Engineering, Auburn University, Auburn, AL 36849, USA; ^3^School of Civil and Environmental Engineering, Georgia Institute of Technology, Atlanta, GA 30332, USA

## Abstract

The photo-Fenton reaction is a promising method to treat organic contaminants in water. In this paper, a Fenton reagent enhanced by microwave electrodeless ultraviolet (MWEUV/Fenton) method was proposed for advanced treatment of nonbiodegradable organic substance in pesticide-containing biotreated wastewater. MWEUV lamp was found to be more effective for chemical oxygen demand (COD) removal than commercial mercury lamps in the Fenton process. The pseudo-first order kinetic model can well describe COD removal from pesticide-containing wastewater by MWEUV/Fenton, and the apparent rate constant (*k*) was 0.0125 min^−1^. The optimal conditions for MWEUV/Fenton process were determined as initial pH of 5, Fe^2+^ dosage of 0.8 mmol/L, and H_2_O_2_ dosage of 100 mmol/L. Under the optimal conditions, the reaction exhibited high mineralization degrees of organics, where COD and dissolved organic carbon (DOC) concentration decreased from 183.2 mg/L to 36.9 mg/L and 43.5 mg/L to 27.8 mg/L, respectively. Three main pesticides in the wastewater, as Dimethoate, Triazophos, and Malathion, were completely removed by the MWEUV/Fenton process within 120 min. The high degree of pesticides decomposition and mineralization was proved by the detected inorganic anions.

## 1. Introduction

The widespread use of pesticides in the past decades represents serious water pollutants [1]. Various types of pesticides residues were frequently detected in surface water and aroused great public concern. The pesticides will cause potential adverse health risks even at low concentration (pg/L to ng/L) [2]. These pesticides, retained in agrochemical wastewater, are resistant to conventional biological treatment owing to their high toxicity and biological persistence. It was reported that no significant decrease in pesticide content occurred after the biological treatment and remaining recalcitrant organic carbon mainly due to pesticide molecules [3].

Advanced oxidation processes (AOPs) were expected to decompose those typically stable products into carbon dioxide, water, and inorganics or, at least, transform them into harmless compounds [4]. AOPs have been successfully applied for the removal of recalcitrant substances from organic wastewater in recent years [5–8]. Among AOPs, photo-Fenton is considered as a promising process to generate oxidizing species for pollutants degradation [9, 10]. Photo-Fenton reaction requires the presence of Fe^2+^ and hydrogen peroxide under the UV radiation to produce highly oxidative hydroxyl radicals which react with the organic pollutants and lead to the complete mineralization [11]. Photo-Fenton is effective in treating wastewater with various organic contaminants, such as dye [12], 2,4-dichlorophenol [13], and nonylphenol polyethoxylate [14]. It has been concluded that the introduction of UV light significantly promotes the degradation efficiency by photoreducing Fe^3+^ to Fe^2+^ and producing additional hydroxyl radicals. Thus, UV light source is a crucial design of photo-Fenton reactor.

Microwave electrodeless ultraviolet (MWEUV) was a new-type UV light source developed in recent years [15, 16]. It includes a mercury vapor lamp powered by microwave energy and emitted stable UV radiation. The MWEUV lamp would lead to a further development of simple and high efficiency photochemical reactor owing to its unique advantages [17–19]: high UV radiant power, long lifetime, and being immersed into reaction solution with adaptable lamp shapes. Particularly, both UV and microwave radiations are available simultaneously by using microwave energy. Many studies have reported successful applications of MWEUV lamp as light source in photolysis [20], UV/H_2_O_2_  [21], and heterogeneous or homogeneous photocatalysis processes [22–24] for organic wastewater treatment. It has been proved that the simultaneous application of microwave power and UV light exhibits higher efficiency in photochemical processes.

In recent years, several advanced oxidation processes for the treatment of pesticide-containing wastewater have been proposed. However, the degradation efficiency for recalcitrant pesticide substances did not meet the demand of commercial application. In this research, the MWEUV lamp was employed for enhancing Fenton process to remove nonbiodegradable organic pesticide in pesticide-containing effluent after biotreatment. Optimal parameters for COD removal by the MWEUV/Fenton process were determined. Oxidation degree and mineralization performance of residual pesticide in wastewater were also evaluated in terms of average oxidation state (AOS), carbon oxidation state (COS), DOC, and inorganic anions concentration.

## 2. Experimental Methodology

### 2.1. Materials and Reagents

Fresh wastewater was generated from pesticide container washing process in a chemical products estate (Guangdong, China). The sequential processes, coagulation, precipitation, and biodegradation, have been employed for elimination of suspended solids and biodegradable organic substances from the pesticide-containing wastewater. Physical/chemical characteristics of fresh wastewater and the biotreated wastewater were showed in Table 1. The concentration of COD and pesticides in effluent wastewater without any advanced treatments still exceeded the discharge limits imposed by Discharge Limits of Water Pollutants (Local Standard: DB44/26-2001).

H_2_O_2_ (30%, w/w) and FeSO_4_·7H_2_O were prepared at a predetermined concentration as Fenton reagent. H_2_SO_4_ (1.0 mmol/L) and NaOH (1.0 mmol/L) were used for pH adjustment. All chemicals were of analytical grade, purchased from Sinopharm Chemical Reagent Co., Ltd (Shenzhen, China).

### 2.2. Experimental Setup and Methods

A U-shaped MWEUV lamp was used in this study. The lamp is made by quartz tubes filled with 1 mg mercury and 0.66 kPa argon. The external diameter is 20 mm and effective length is 250 mm. The MWEUV lamp had prominent UV emission bands at 254, 313, 365, and 405 nm. Microwave generator (Haier Co. Ltd, China) was operated with 80 W output at frequency of 2.45 GHz. The output power of MWEUV lamp was measured by monitoring the temperature of the solution with and without the MWEUV lamp [25]. According the calculation, approximate 40 W was supplied to excite MWEUV lamp and the rest power was consumed or converted to heat.

**Figure 1:**
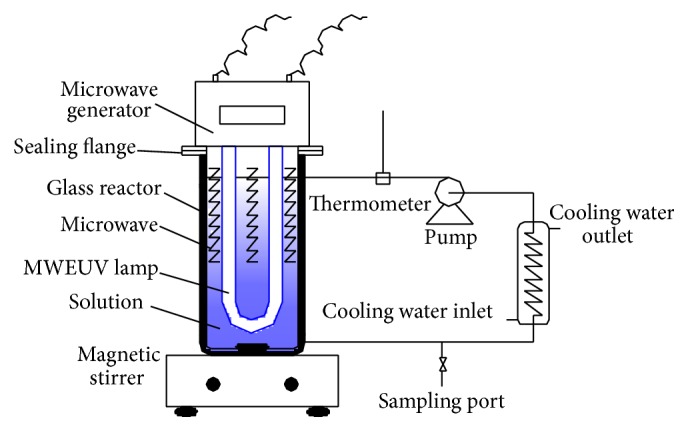
Schematic diagram of experimental setup.

Schematic diagram of the experimental setup is shown in Figure 1. 1000 mL pesticide-containing wastewater was added in the glass cylindrical reactor (available capacity of 1000 mL). Then, required dosage of Fenton reagent was added into the solution and mixed by a magnetic stirrer (HJ-3, Jingda Instrument Company, China). Microwave generator was applied to excited MWEUV lamp emitting UV irradiation during the reaction. Solution temperature during the reaction was kept constant at 25 ± 0.5°C by circulating solution to a cooler with a pump (DP-60, Seisun pumps Co., Ltd, China). Samples were withdrawn at predetermined time intervals, and the final pH values were adjusted to lower than 3 before analysis. All the runs were triplicated.

In this study, the experiments were carried out as follows and the results were compared: (1) Fenton oxidation run, (2) UV/Fenton run, assisted by a commercial UV mercury lamp (40 W of output, CREATOR, China), and (3) MWEUV/Fenton run, assisted by MWEUV lamp.

### 2.3. Analytical Methods

The pH value of solution was measured using a pH meter (PHB-5, INESA Instrument, China). Total suspended solids (TSS) were measured by gravimetry method. Chemical oxygen demand (COD) was determined according to the Standard Methods [26]. Dissolved organic carbon (DOC) was measured by TOC analyzer (Multi N/C, Jena). NO_3_^−^, SO_4_^2−^, and PO_4_^3−^ were quantified by ion chromatography (Dionex ICS-900; column Ion Pac AS23; suppressor MMS 300). Isocratic elution was done with KOH solution, at a flow rate of 1.0 mL/min, for anions analyzed. The quantitative analysis of pesticides was performed by GC/MS (Agilent, 6890N-5975B). Samples were subsequently injected in GC, coupled with a mass spectrometer. The mass detector was operated in MRM (Multiple Reaction Monitoring) mode, selecting specific transitions for each pesticide. The final optimized method allowed the concurrent detection of three pesticides, during chromatographic runs of 20 min.

## 3. Results and Discussion

### 3.1. Evaluation of Different Treatment Processes

Photo-Fenton process produces hydroxyl radicals with powerful oxidizing ability to degrade organic pollution. The major reactions to form hydroxyl radicals are as follows [5]:

(1) Fe2++H2O2Fe3++∙OH+OH−

(2) Fe3++H2O⟶FeOH2++H+

(3) FeOH2+⟶hνFe2++∙OH

(4) H2O2⟶hν∙OH+∙OH

**Figure 2:**
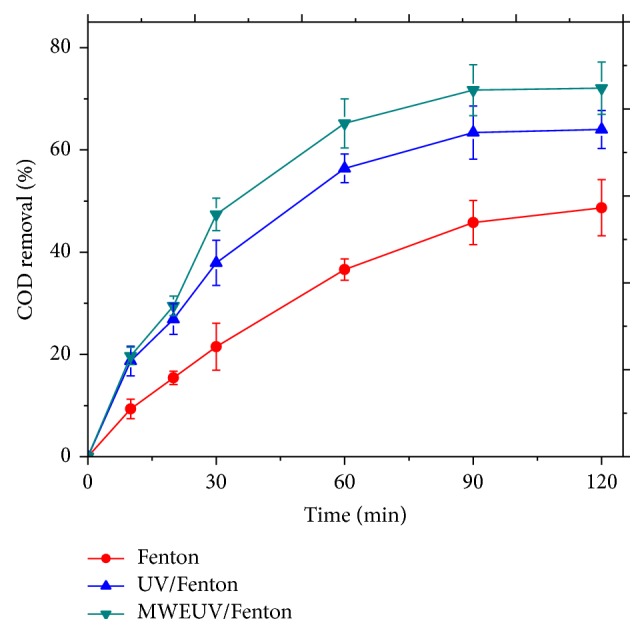
COD removal in different treatment processes. Experimental conditions: initial pH value of 5, H_2_O_2_ dosage of 40 mmol/L, and Fe^2+^ dosage of 0.6 mmol/L.

In this study, treatment of pesticide-containing wastewater in different processes was evaluated. Specially, Fenton oxidation assisted by MWEUV and a commercial mercury UV lamp was compared. As shown in Figure 2, Fenton oxidation only achieved 48.7% of COD removal after 120 min, while COD removal efficiency was significantly improved by introducing UV light. The COD concentration of pesticide-containing wastewater with UV/Fenton treatment decreased 64.0%. Such improvement was attributed to two facts: on the one hand, the reduction of Fe^3+^ (Equations (2), (3)) under UV irradiation regenerated Fe^2+^ that can further participate in the Fenton reaction and produce ∙OH radicals. Such recycling enhanced the ∙OH radical production and provides the UV/Fenton system sufficient activity for organic molecule decomposition. On the other hand, photolysis of H_2_O_2_ (Equation (4)) generates more OH radicals. It is also notable that MWEUV lamp as the light source is more effective in the Fenton system than a commercial mercury lamp. 72.1% of COD in wastewater was removed by MWEUV/Fenton treatment within 120 min. In this experiment, wastewater temperature during the reaction was kept constant. It means that microwave irradiation accelerates chemical reactions by nonthermal effects such as polarization, dielectric properties, nuclear spin rotation, and spin alignment [27]. It was reported that simultaneous irradiation of microwave and UV-Vis light led to effective reactant decomposition by microwave ultraviolet electrodeless lamp introduced in photolysis, photocatalysis, and UV/H_2_O_2_ systems [25, 27, 28].

### 3.2. Effect of Initial pH Value

**Figure 3:**
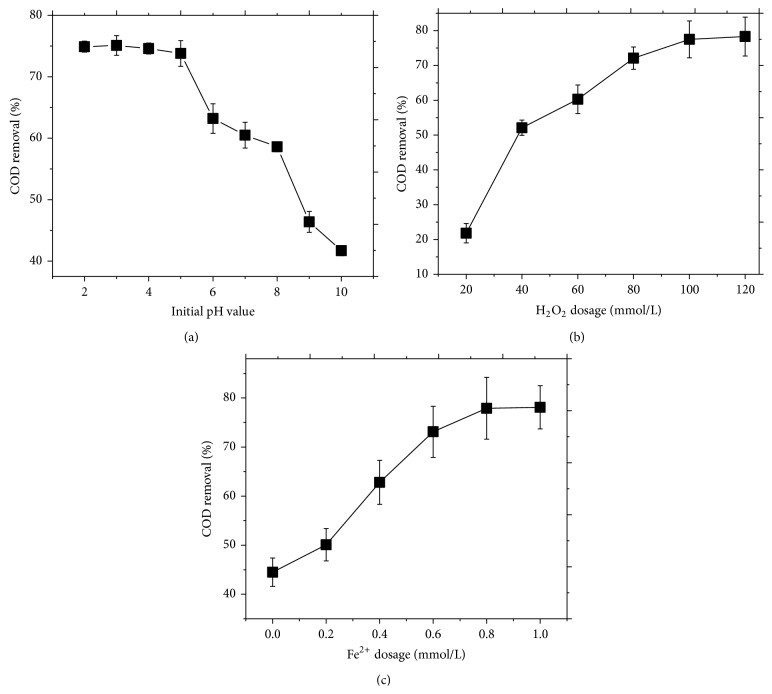
Effect of (a) initial pH value with H_2_O_2_ dosage of 40 mmol/L, (b) H_2_O_2_ dosage, and (c) Fe^2+^ dosage with H_2_O_2_ dosage of 100 mmol/L on COD removal. General experimental parameters: initial pH value of 5, Fe^2+^ dosage of 0.6 mmol/L, and reaction time of 120 min.

The pH of wastewater is an important parameter for the MWEUV/Fenton process because it affects the decomposition of hydrogen peroxide [29] and the hydrolytic speciation of the ferric ion species [30]. To test the effect of initial pH, experiments were carried out at initial pH ranging from 2 to 10; the results of COD removal by MWEUV/Fenton are shown in Figure 3(a). It is clear that removal efficiency is highly dependent on initial pH, and the optimum pH is between 2 and 4. The COD removal efficiency decreased from 73.8% to 41.7% according to the increase of pH from 5 to 9. Many researchers have also reported the optimum pH as 3–5 for conventional Fenton or photo-Fenton processes [31–33]. Neamtu et al. [34] summarized various photoactive species of iron formed at different pH conditions. The dominant species at pH 1-2 is Fe[H_2_O]_6_^3+^, 2-3 is for Fe[OH][H_2_O]_5_^2+^, and 3-4 is for Fe[OH]_2_[H_2_O]_4_^+^. Fe(OH)^2+^ species is reported to have the highest photoreactivity in Fenton's oxidation [35]. At acidic pH, Fe^3+^ hydroxyl complexes are highly soluble and Fe(OH)^2+^ is the predominant form of the Fe^3+^ hydroxyl complexes. At higher pH, the concentration of Fe[OH]^2+^ complex ions in solution decreased with the precipitation of ferrous ion as oxyhydroxides [36]. Under the alkaline solution condition, the removal of pollutant is mainly attributed to adsorption and coagulation rather than Fenton oxidation [37].

### 3.3. Effect of H_2_O_2_ Dosage

Effect of H_2_O_2_ dosage on COD removal from pesticide-containing wastewater by MWEUV/Fenton is shown in Figure 3(b). As expected, the increase of H_2_O_2_ dosage from 20 to 80 mmol/L accelerated COD removal. The increased degradation efficiency can be attributed to the additional OH radicals produced from H_2_O_2_ decomposition. However, COD removal could not be obviously improved by excessive addition of H_2_O_2_ (>100 mmol/L). The results showed a negligible increase from 77.5% to 78.3% in removal efficiency when H_2_O_2_ dosage further increased from 100 to 120 mmol/L. It can be interpreted that the excessive H_2_O_2_ acts as a scavenger of ∙OH, but the produced HO_2_∙ (Equations (5)–(7)) has much lower oxidation capacities [38]. Therefore, the optimum H_2_O_2_ dosage was found to be 100 mmol/L for advanced treatment of the pesticide-containing wastewater by the MWEUV/Fenton:

(5) H2O2+∙OHHO2∙+H2O

(6) HO2∙+OH⟶H2O+O2

(7) ∙OH+∙OH⟶H2O2

Li et al. [39] investigated catechol oxidation in nano-Fe_3_O_4_ catalyzing UV-Fenton process. The best operational H_2_O_2_ dosage was determined to obtain high efficiency of both H_2_O_2_ utilization and COD removal. Lucas and Peres [40] observed the decreasing dye decolorization at excessive H_2_O_2_ concentration (>2 mmol/L) for Reactive Black 5 in Fenton/UV-C and ferrioxalate/H_2_O_2_/solar light processes. Zhong et al. [41] also indicated that the degradation efficiency of tetrabromobisphenol A significantly decreased in heterogeneous UV/Fenton with the H_2_O_2_ concentration increasing to 20 mmol/L.

### 3.4. Effect of Fe^2+^ Dosage

To investigate effect of Fe^2+^ dosage on COD removal, experiments were carried out at Fe^2+^ dosage ranging from 0 to 1.0 mmol/L.  Figure 3(c) showed a significant increase in COD removal efficiency from 44.5% to 77.9% with the increasing Fe^2+^ concentration 0 to 0.8 mmol/L. Fe^2+^ is important for formation of photoactive ferric-hydroxo complexes that absorb UV light to produce ∙OH [42]. However, further increases in Fe^2+^ concentration up to 0.8 mmol/L only resulted in slight increases in degradation rate. Excessive ferrous ions may act as hydroxyl radical scavenger according to the following [43]:

(8) $$\begin{array}{c} \text{F}{\text{e}}^{2+}+∙\text{OH}⟶\text{F}{\text{e}}^{3+}+\text{O}{\text{H}}^{-} \end{array}$$

In addition, a deep color and high turbidity at high Fe^2+^ concentration reduced the transmission of UV light in solution, which inhibited the photolysis of H_2_O_2_ to produce OH radicals [31]. Therefore, overdosed Fe^2+^ was inefficient for COD removal by MWEUV/Fenton process.

### 3.5. Kinetics of COD Removal by MWEUV/Fenton

**Figure 4:**
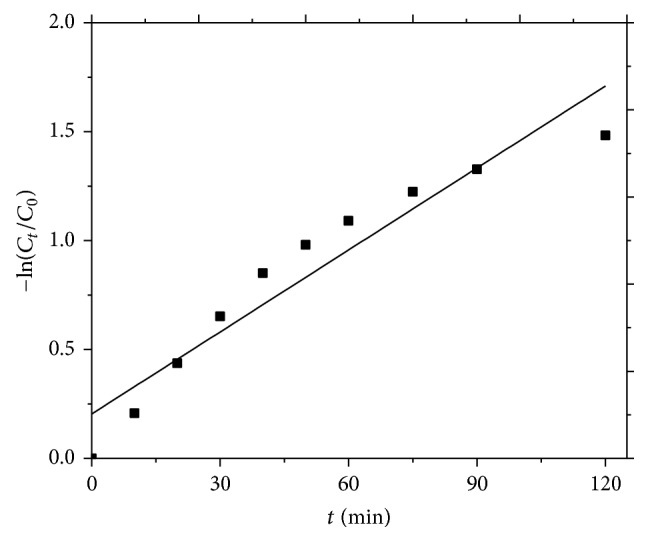
Kinetics of COD decomposition by MWEUV/Fenton. Experimental parameters: initial pH value of 5, H_2_O_2_ dosage of 100 mmol/L, and Fe^2+^ dosage of 0.8 mmol/L.

Experiments were conducted under the optimum operating conditions to evaluate the kinetics of MWEUV/Fenton degradation for pesticide-containing wastewater. A simple pseudo-first order kinetic model was used to fit experimental data and determine the kinetic parameters:

(9) $$\begin{array}{c} \frac{-\ln C_{t}}{C_{0}}=kt, \end{array}$$

where *C*_*t*_ is COD concentration (mg/L) at time *t* (min), *C*_0_ is initial COD concentration (mg/L), and *k* is apparent rate constant (min^−1^). A plot of −ln(*C*_*t*_/*C*_0_) versus *t* generates a straight line. Apparent rate constant for the COD degradation was determined from the slope of the straight line. From Figure 4, COD degradation in MWEUV/Fenton followed pseudo-first order kinetics with rate constants (*k*) of 0.0125 min^−1^ and *R* ^2^ (correlation coefficient) of 0.9171.

### 3.6. Evaluation of Oxidation Degree and Mineralization

At present, two parameters, as AOS and COS, are defined to evaluate the oxidation degree and oxidative process efficiency, respectively [3]:

(10) AOS4−1.5CODtDOCt,

(11) COS=4−1.5CODtDOC0,

where COD_*t*_ is the chemical oxygen demand (mg/L) at time *t* (min), COD_*t*_ is the dissolved organic carbon (mg/L) at time *t*, and DOC_0_ is the initial dissolved organic carbon (mg/L). AOS takes values between +4 for CO_2_, the most oxidized state of C, and −4 for CH_4_, the most reduced state of C.

**Figure 5:**
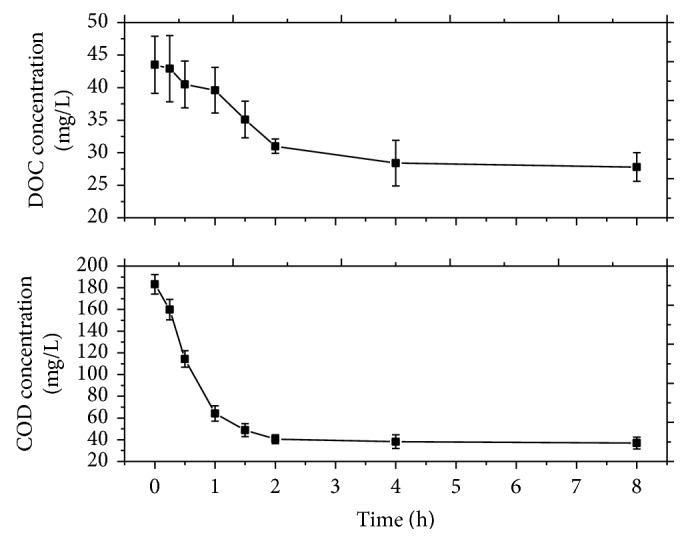
Evolution of COD and DOC in MWEUV/Fenton. Experimental parameters: initial pH value of 5, H_2_O_2_ dosage of 100 mmol/L, and Fe^2+^ dosage of 0.8 mmol/L.

**Figure 6:**
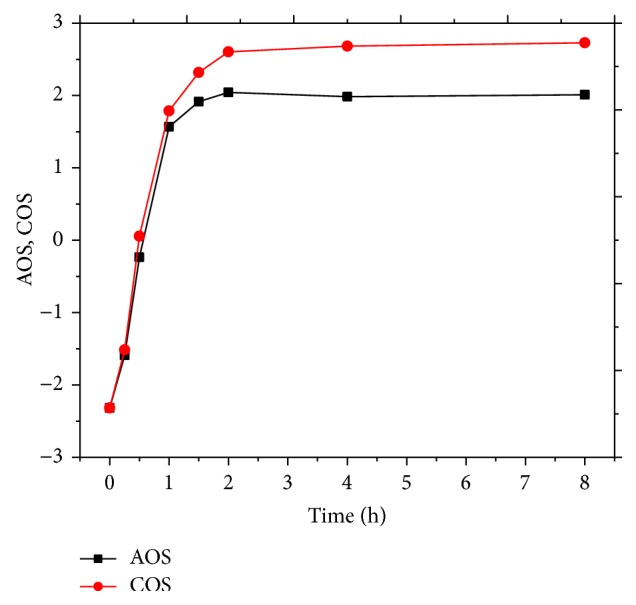
Evolution of AOS and COS in MWEUV/Fenton. Experimental parameters: initial pH value of 5, H_2_O_2_ dosage of 100 mmol/L, and Fe^2+^ dosage of 0.8 mmol/L.

Figures 5 and 6 present the evolution of COD, DOC, AOS, and COS with reaction time. Both the initial AOS and COS parameters were −2.32, indicating that organic compounds are at reduced state in wastewater. With the prolonging of reaction time, the concentrations of COD and DOC in wastewater gradually decreased, showing a strong oxidation of the organics. AOS and COS parameters increased to be positive, suggesting that strong mineralization occurred and highly oxidized intermediates are generated. Analyzing the different process phases, more oxidized organic intermediates were formed before 60 min of reaction without substantial mineralization, which is corroborated by rapid COD decrease and low DOC removal. After the gradual growth, the values of AOS and COS reached the plateau. Finally, the concentrations of COD and DOC decreased to 36.9 mg/L and 27.8 mg/L, respectively, with the AOS value of 2.73 and the COS value of 2.01. The results indicate that a high oxidation degree of pesticides related to the generation of some low molecular weight carbohydrates which are resistant to mineralization by hydroxyl radicals.

**Figure 7:**
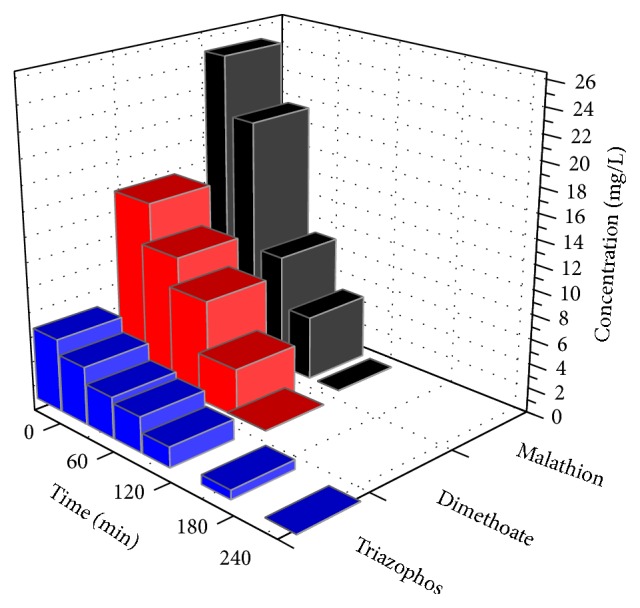
Degradation of Dimethoate, Triazophos, and Malathion in MWEUV/Fenton. Experimental parameters: initial pH value of 5, H_2_O_2_ dosage of 100 mmol/L, and Fe^2+^ dosage of 0.8 mmol/L.

**Figure 8:**
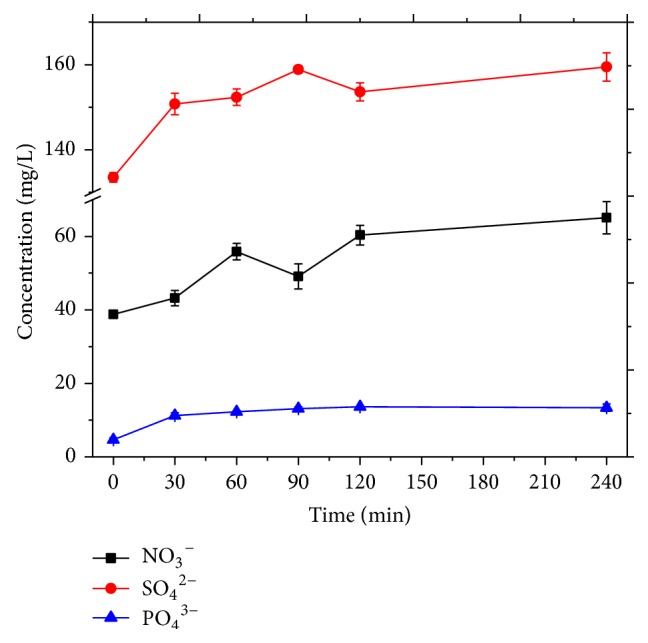
Evolution of inorganic anions in MWEUV/Fenton. Experimental parameters: initial pH value of 5, H_2_O_2_ dosage of 100 mmol/L, and Fe^2+^ dosage of 0.8 mmol/L.

The concentration profiles of Dimethoate, Triazophos, and Malathion during the MWEUV/Fenton process are shown in Figure 7. They displayed similar profiles, a rapid increase in pesticides concentration at the initial stage of MWEUV/Fenton process, and Dimethoate and Malathion were completely removed within 240 min.  Figure 8 exhibits the concentration changes of inorganic anions (NO_3_^−^, SO_4_^2−^, and PO_4_^3−^) in the reaction. The increases of SO_4_^2−^ and PO_4_^3−^ concentration in wastewater were mainly attributed to the breakage of chemical bonds such as S=P, P-O, and S-P. The results also suggested a deep decomposition and mineralization of pesticide molecules occurring under the attack of hydroxyl radicals.

## 4. Conclusions 

The feasibility and superiority of the MWEUV/Fenton for advanced treatment of pesticide-containing biotreated wastewater were evaluated in this paper. In terms of COD removal, the MWEUV/Fenton process showed high efficiency comparing with the conventional Fenton process assisted by commercial mercury lamps. The optimal parameters were found at the initial pH of 5, Fe^2+^ dosage of 0.8 mmol/L, and H_2_O_2_ dosage of 100 mmol/L. Three main pesticides, Dimethoate, Triazophos, and Malathion, in the wastewater were completely decomposed with high oxidation and mineralization degrees.

## Figures and Tables

**Table 1:** Physical/chemical characteristics of fresh wastewater and the biotreated wastewater.

Concentration (mg/L)	Fresh wastewater	Effluent after biological treatment
pH	6.0~6.8	~7.0
TSS	618.85 ± 70.90	<15
COD	1540.34 ± 202.12	183.2 ± 9.95
BOD_5_	336.58 ± 20.86	<15
Dimethoate (C_5_H_12_NO_3_PS_2_)	15.66 ± 3.38	14.71 ± 0.79
Triazophos (C_12_H_16_N_3_O_3_PS)	6.11 ± 0.63	5.87 ± 0.89
Malathion (C_10_H_19_O_6_PS_2_)	31.65 ± 4.77	24.53 ± 4.16
